# Liquid L-T4 therapy in hypothyroid patients with gastric diseases, an observational study

**DOI:** 10.3389/fendo.2024.1386629

**Published:** 2024-07-04

**Authors:** Poupak Fallahi, Francesca Ragusa, Armando Patrizio, Valeria Mazzi, Chiara Botrini, Giusy Elia, Eugenia Balestri, Emilio Barozzi, Licia Rugani, Elena Palmisano, Maria Carla Cosenza, Gilda Varricchi, Salvatore Ulisse, Salvatore Benvenga, Silvia Martina Ferrari, Alessandro Antonelli

**Affiliations:** ^1^ Department of Translational Research and New Technologies in Medicine and Surgery, University of Pisa, Pisa, Italy; ^2^ Department of Surgical, Medical and Molecular Pathology and Critical Area, University of Pisa, Pisa, Italy; ^3^ Department of Emergency Medicine, Azienda Ospedaliero-Universitaria Pisana, Pisa, Italy; ^4^ Department of Translational Medical Sciences and Center for Basic and Clinical Immunology Research (CISI), University of Naples Federico II, Naples, Italy; ^5^ World Allergy Organization (WAO) Center of Excellence, Naples, Italy; ^6^ Institute of Experimental Endocrinology and Oncology (IEOS), National Research Council, Naples, Italy; ^7^ Department of Surgery, “Sapienza” University of Rome, Rome, Italy; ^8^ Department of Clinical and Experimental Medicine, University of Messina, Messina, Italy; ^9^ Master Program on Childhood, Adolescent and Women’s Endocrine Health, University of Messina, Messina, Italy; ^10^ Interdepartmental Program of Molecular and Clinical Endocrinology and Women’s Endocrine Health, Azienda Ospedaliera Universitaria Policlinico “G. Martino”, Messina, Italy; ^11^ Department of Clinical and Experimental Medicine, University of Pisa, Pisa, Italy

**Keywords:** levothyroxine, liquid L-T4, hypothyroidism, gastritis, gastrectomy, gastroplastic, gastric disease

## Abstract

**Introduction:**

This is an observational and retrospective study, in which we have analyzed data from patients affected by gastric diseases (p) who have been treated with liquid L-T4 (L-LT4;84 p), or tablet L-T4 (T-LT4;120 p), for the replacement therapy of hypothyroidism. The aim of the study is to compare the stability of TSH [normal range, 0.3-3.5 μIU/ml] in these patients.

**Methods:**

All p assumed L-T4 30 minutes before breakfast. The types of gastric disease were: a) T-LT4 group: 74 chronic gastritis (CG); 4 gastrectomy for gastric cancer (GTx); 42 gastro-plastics (GP); b) L-LT4 group: 60 CG; 3 GTx; 21 GP (p>0.05). 66% p in T-LT4 group were chronically treated with proton pump inhibitors (PPI), against 51% in L-LT4 group (p>0.05). The frequency of Helicobacter Pylori infection was 17% in both T-LT4 and L-LT4 groups. The gender distribution, mean age and body weight were similar in the 2 groups (p>0.05). The mean L-T4 dosage in T-LT4 group at the basal evaluation was 1.22+/-0.27 μg/kg/die, in the L-LT4 group 1.36+/-0.22 μg/kg/die (p>0.05).

**Results:**

At the basal evaluation the prevalence of patients with a TSH>3.5 μIU/mL in T-LT4 group was 36%, in L-LT4 group 46% (p<0.05). After adjustment of the dosage of the LT-4 therapy, the p were re-evaluated in an interval range of 5-9 months, for 4 times, during an overall period ranging from 23 to 31 months. At the first re-evaluation, the prevalence of p with a TSH>3.5 μIU/mL was 13% in both groups. At the second re-evaluation, the prevalence of p with a TSH>3.5 μIU/mL in T-LT4 group was 26%, in L-LT4 group 13% (p>0.05). At the third re-evaluation, the prevalence of p with TSH<3.5 μIU/mL in T-LT4 group was 19%, in L-LT4 group 9% (p=0.05). At the fourth and last re-evaluation, the prevalence of patients with a TSH>3.5 μIU/mL in T-LT4 group was 18%, in L-LT4 group 5% (p<0.05). Mean FT4 and FT3 circulating levels were not significantly different in the two group at each visit.

**Discussion:**

These data suggest that the liquid L-T4 formulation therapy can result in a more stable control of TSH levels in hypothyroid patients with gastric disorders in the long-term follow-up.

## 1 Introduction

The cornerstone for the treatment of all forms of hypothyroidism, primary, central and iatrogenic, has been represented for a long time by Levothyroxine (L-T4) tablets (1–3). This type of formulation is absorbed, with an average bioavailability of 70%, in the first section of the small intestine, duodenum and jejunum, after a correct digestive process that takes place in the stomach, called gastric dissolution (1–4). Here the tablet, which is composed of sodium L-T4 together with other excipients, is converted into an absorbable lipophilic compound by the acidic environment (5, 6). Therefore, any endogenous or exogenous factor, able to alter the gastric pH, can impair the complete absorption of L-T4 tablets resulting in TSH levels higher than desired for that specific patient and subsequent therapeutic failure. In 2006, Centanni et al. demonstrated for the first time how chronic gastric diseases, such as Helicobacter Pylori infection and atrophic gastritis, prevents the correct absorption of L-T4 tablets, resulting in higher TSH levels, partially reversible by the infection eradication (5). Since then, many studies have shown that this phenomenon also occurs in the case of other gastric diseases such as autoimmune gastritis (7, 8) or with the co-administration of drugs that alter the gastric pH [i.e. proton pump inhibitors (PPI), Calcium and iron supplements] (9–12), which are very commonly prescribed among hypothyroid patients. An important turning point came with the introduction on the market of the new oral formulations made of L-T4 in liquid solution (13–17), which does not require the gastric dissolution phase and reduces the risk of LT4 malabsorption.

The specific aim of this observational and retrospective study was to evaluate the stability of TSH [TSH in the normal range, 0.3–3.5 µIU/ml] and thyroid hormone levels in hypothyroid patients with gastric disease treated with different formulations of L-T4 [liquid (L-LT4), or tablet (T-LT4)].

## 2 Methods

The data used for this study were routinely collected from patients during their visits to our clinical site as recorded in the medical records. Data were abstracted from the medical records and entered into a database from patients who met the following characteristics: 1- subjects aged ≥ 18 years being treated with L-T4 for hypothyroidism, due to autoimmune thyroiditis, or after partial or total thyroidectomy, in our center from January 2013 to August 2021, followed for at least 2 years and with at least two TSH determinations in the 24-month evaluation period; 2- subjects with an established diagnosis of a gastric disease or disorder; 3- subjects taking the therapy correctly (in the morning, following an overnight fast, 30 minutes before the consumption of food or liquids (excluding water) or other drugs).

All patients (meeting the above-mentioned criteria) who started with liquid formulation of L-T4 from the basal evaluation (regardless the previous management), have been included in the study. They have been matched by age and sex, with patients treated with the L-T4 tablet with gastric disorders in the same period.

The diagnosis of gastric diseases was performed along standard methods.

The diagnosis of chronic gastritis was performed in patients with dyspeptic symptoms with the histological examination of the bioptic samples of the gastric mucosa obtained with gastroscopy; patients were also evaluated for the presence of anti-parietal cells autoantibodies, H. pylori infection, and vitamin B12 deficiency (18). Furthermore, to avoid bias in the evaluation of T4 malabsorption, only patients without reported symptoms and with negative results for the following tests were included in the analysis: a) lactose tolerance test; b) anti-tissue transglutaminase IgG and IgA antibodies, gastrin, antiendomysial IgA and IgG antibodies (19). Other conditions associated with L-T4 malabsorption were not present, as: a) concurrent therapy with amiodarone, beta-blockers, orlistat, lithium, raloxifen, interferons, cholestyramine (5, 11); b) preceding intestinal surgery. Moreover, we excluded pseudo-malabsorption owing to poor compliance in each patient.

The Investigator sought for written informed consent to the data analysis from all subjects. The study was conducted in accordance with the Declaration of Helsinki, and the protocol was approved by the local Ethical Committee (Comitato Etico di Area Vasta Nord Ovest per lo studio ID n. 16596/2020).

All patients were treated with liquid L-T4 (Tirosint, IBSA Farmaceutici Italia Srl, Lodi, Italy, containing as excipients: 96% ethanol; 85% glycerol) (L-LT4 group), or the L-T4 tablets (from different pharmaceutical companies; T-LT4 group), administered 30 minutes before breakfast.

After the initial evaluation (basal) the L-T4 dose was adjusted if needed and, the patients were re-evaluated in an interval range of 5–9 months, for 4 times, during an overall period ranging from 23 to 31 months in both groups. During each re-evaluation the therapy was adjusted on the base of TSH, FT4, and FT3 levels, in all patients.

At the basal evaluation and during the 4 control examinations circulating levels of FT4 (normal range, 0.7–1.7 ng/dL), FT3 (normal range, 2.7–4.7 pg/mL), and TSH (normal range, 0.3–3.5 μIU/mL) were tested.

### 2.1 Data analysis

Values are given as mean ± SD for normally distributed variables (evaluated by the Shapiro-Wilk test; P < 0.05 was considered as statistically significant), alternatively as median and [interquartile range]. One-way analysis of variance (ANOVA) was performed to compare mean group values (for normally distributed variables, such as body weight and age). Chi-Square test was done to compare the proportions. Statistical analysis was conducted using STATA, and StatView.

The correlation among changes in TSH was evaluated by simple regression at the basal evaluation vs. FT4 or FT3.

## 3 Results

### 3.1 Basal evaluation

The gender distribution was similar in tablet L-T4 (T-LT4) treated patients (120; 95 females, 79.2%; 25 males, 20.8%) with respect to liquid LT-4 (L-LT4) treated patients (84: 68 females, 80%; 16 males, 20%) (p>0.05). The mean age distribution was non significantly different between two groups (T-LT4: 55.9 ys +/- 15.6; L-LT4: 54.5 ys +/- 15.9; p>0.05). Also basal body weight was not significantly different between the two groups (T-LT4: 70.5 +/- 16.9 Kg; L-LT4: 69.6 +/- 15.1 Kg; p>0.05) (**Table 1**).

**Table 1:** Characteristics of patients treated with tablet-L-T4, or liquid-L-T4.

	Tablet	Liquid	P
**Gender (M/F)**	25/95	16/68	p>0.05
**Age** **[Years; Mean (SD)]**	55.96 (15.61)	54.51 (15.96)	p>0.05
**Basal body weight** **[Kg; Mean (SD)]**	70.54 (16.96)	69.63 (15.07)	p>0.05
**Type of gastric disease**	74 chronic gastritis 4 gastrectomy for gastric cancer 42 gastroplastic	60 chronic gastritis 3 gastrectomy for gastric cancer 21 gastroplastic	p>0.05
**PPI treatment (n° of patients)**	79	43	p=0.051
**Frequency of HP infection**	17%	17%	p>0.05
**Starting mean LT4 dose (μg/kg/die)**	1.22 +/- 0.23	1.36 +/- 0.27	p>0.05

F, females; M, males.

The types of gastric disease were as following: a) in T-LT4 group: 74 chronic gastritis; 4 gastrectomy for gastric cancer; 42 gastro-plastics; b) in L-LT4 group: 60 chronic gastritis; 3 gastrectomy for gastric cancer; 21 gastro-plastics; the frequency distribution is not significantly different between the two groups (p>0.05).

Seventy-nine patients (66%) in the T-LT4 group were chronically treated with PPI, against 43 (51%) patients in the L-LT4 group (Yates corrected chi-square, p=0.051).

The frequency of Helicobacter Pylori infection was the same in two groups (17% in both T-LT4 and L-LT4 groups; p>0.05).

**Figure 1:**
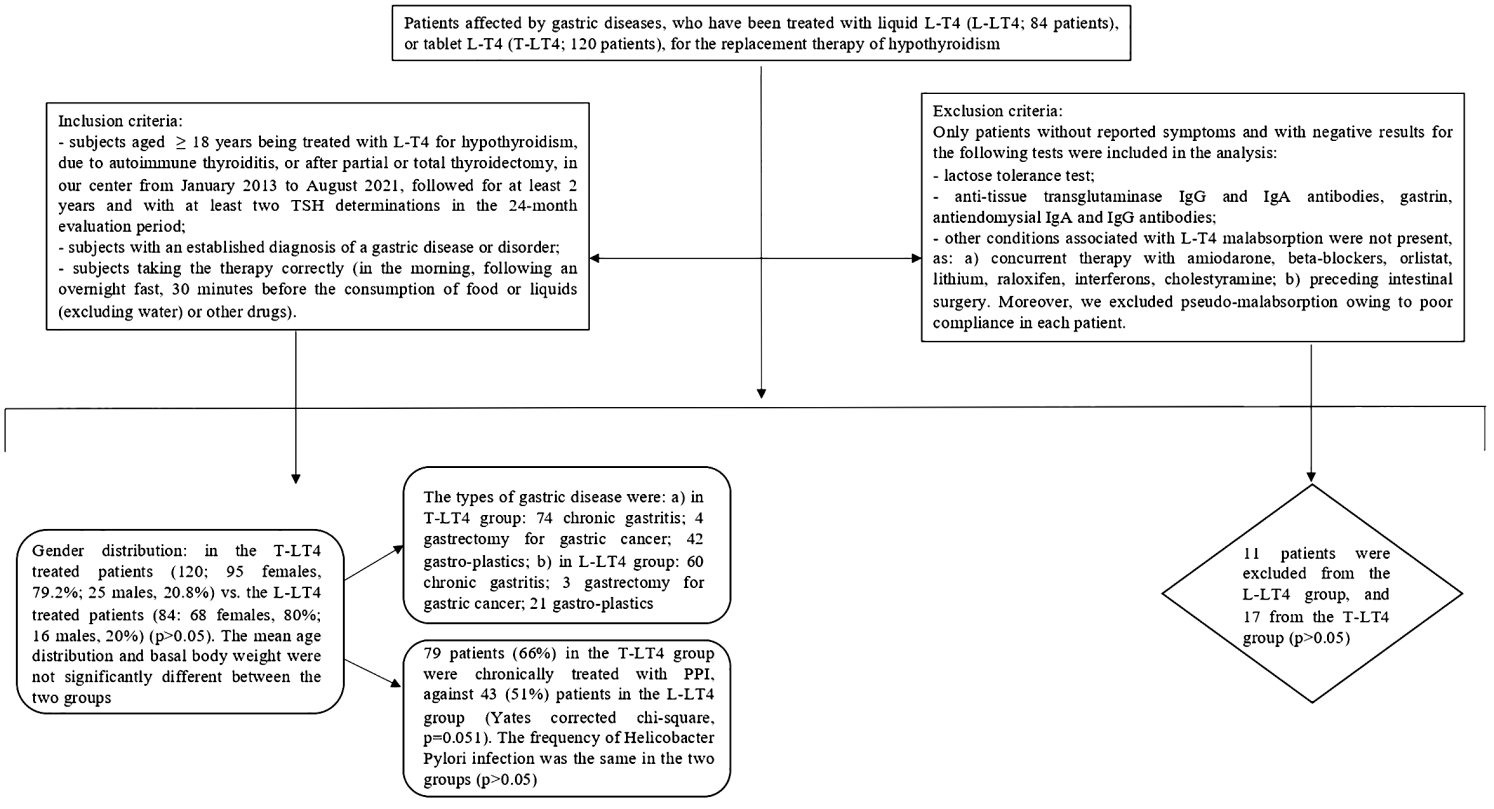
Flow-chart of the study.

On the whole, we excluded 11 patients from the L-LT4 group, and 17 from the T-LT4 group (p>0.05) (**Figure 1** **).**

The starting mean L-T4 dosage in the T-LT4 group that was given to the patients after the basal evaluation was 1.22 +/- 0.23 μg/kg/die; while in the L-LT4 group 1.36 +/- 0.27 μg/kg/die (p>0.05 by ANOVA).

**Figure 2:**
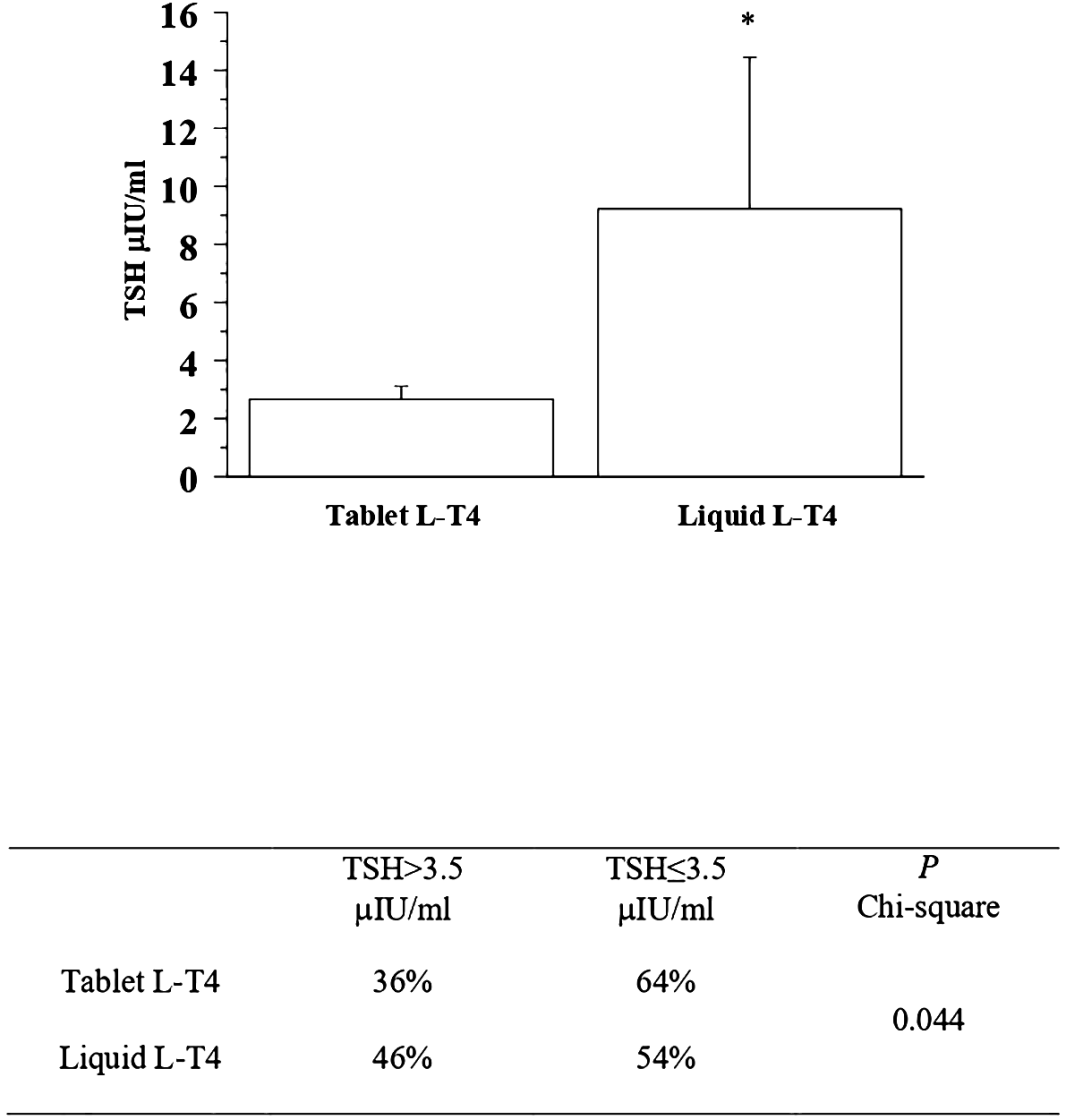
The prevalence of patients with a TSH higher than 3.5 μIU/mL in the T-LT4 group was 36% that was significantly lower than the prevalence in the L-LT4 group (46%) at the basal evaluation. Also mean TSH level (Mean+/- SE) was significantly higher in the L-LT4 group with respect to T-LT4 group (ANOVA *p value = 0.05).

At the basal evaluation the prevalence of patients with a TSH higher than 3.5 μIU/mL in the T-LT4 group was 36% that was significantly lower than the prevalence in the L-LT4 group (46%) (see **Figure 2**, **Table 2**). Also mean TSH level was significantly higher in the L-LT4 group with respect to T-LT4 (see **Figure 2**). The prevalence of TSH levels >10 μIU/ml was 4.09% in the T-LT4 group versus 9.87% in the L-LT4 group (chi-square, p=0.099). The prevalence of patients with TSH<0.3 μIU/ml was 10.3% in the T-LT4 group, against 5.9% in the L-LT4 group (chi-square, p=0.65). Mean FT4 and FT3 circulating levels were not significantly different in the two groups.

**Table 2:** Prevalence of patients with TSH > 3.5 μIU/mL or TSH < 3.5 μIU/mL in the two groups of patients (T-LT4 or L-LT4 group) at each time-point of evaluation.

Time-point of evaluation		TSH>3.5 μIU/ml	TSH ≤ 3.5 μIU/ml	P (chi-square)
Basal evaluation	Tablet L-T4	36%	64%	0.044
	Liquid L-T4	46%	54%	
First re-evaluation	Tablet L-T4	13%	87%	0.925
	Liquid L-T4	13%	87%	
Second re-evaluation	Tablet L-T4	26%	74%	0.030
	Liquid L-T4	13%	87%	
Third re-evaluation	Tablet L-T4	19%	81%	0.051
	Liquid L-T4	9%	91%	
Fourth and last re-evaluation	Tablet L-T4	18%	82%	0.027
	Liquid L-T4	5%	95%	

A simple regression showed a negative correlation between the TSH values vs. FT4 at the basal evaluation in both groups (T-LT4, r = 0.654, p = 0.02; L-LT4, r = 0.721, p = 0.01). Moreover, no significant association was reported between FT3 and TSH in both groups at the basal evaluation (p > 0.05, for both).

### 3.2 Follow-up evaluations

**Figure 3:**
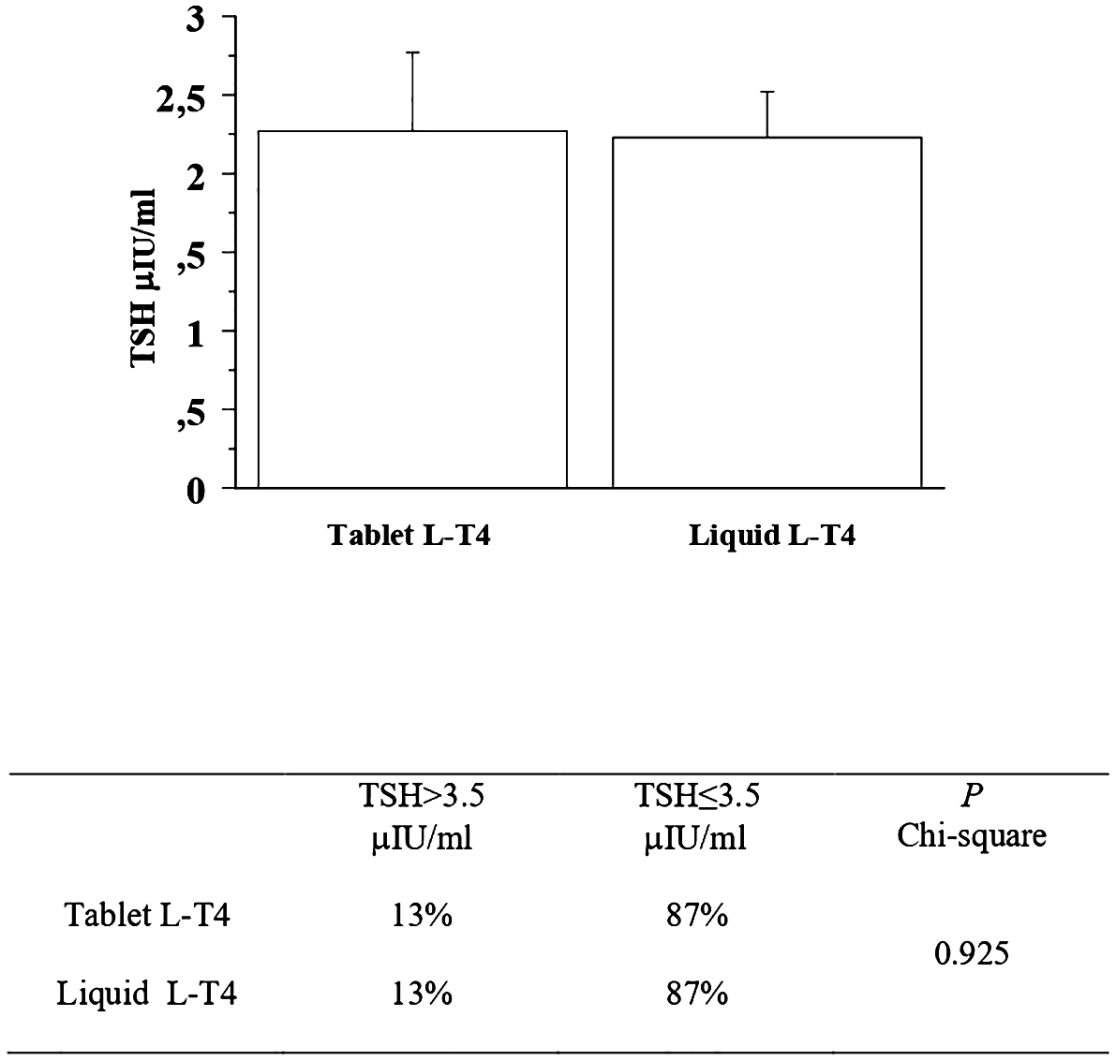
At the first re-evaluation, the prevalence of patients with a TSH higher than 3.5 μIU/mL in the T-LT4 group was 13% that was quite similar to the prevalence in the L-LT4 group (13%). Also mean TSH levels were similar in the two groups (ANOVA p value = 0.95).

At the first re-evaluation, the prevalence of patients with a TSH higher than 3.5 μIU/mL in the T-LT4 group was 13% that was equal to the prevalence in the L-LT4 group (13%) (see **Figure 3**, **Table 2**). Also mean TSH levels were similar in the two groups (see **Figure 3**). The prevalence of TSH levels >10 μIU/mL was 1.6% in the T-LT4 group, versus 2.5% in the L-LT4 group (chi-square, p=0.65). The prevalence of patients with TSH<0.3 μIU/ml was 13.9% in the T-LT4 group against 12.6% in the L-LT4 group (chi-square, p=0.79). Mean FT4 and FT3 circulating levels were not significantly different in the two groups.

**Figure 4:**
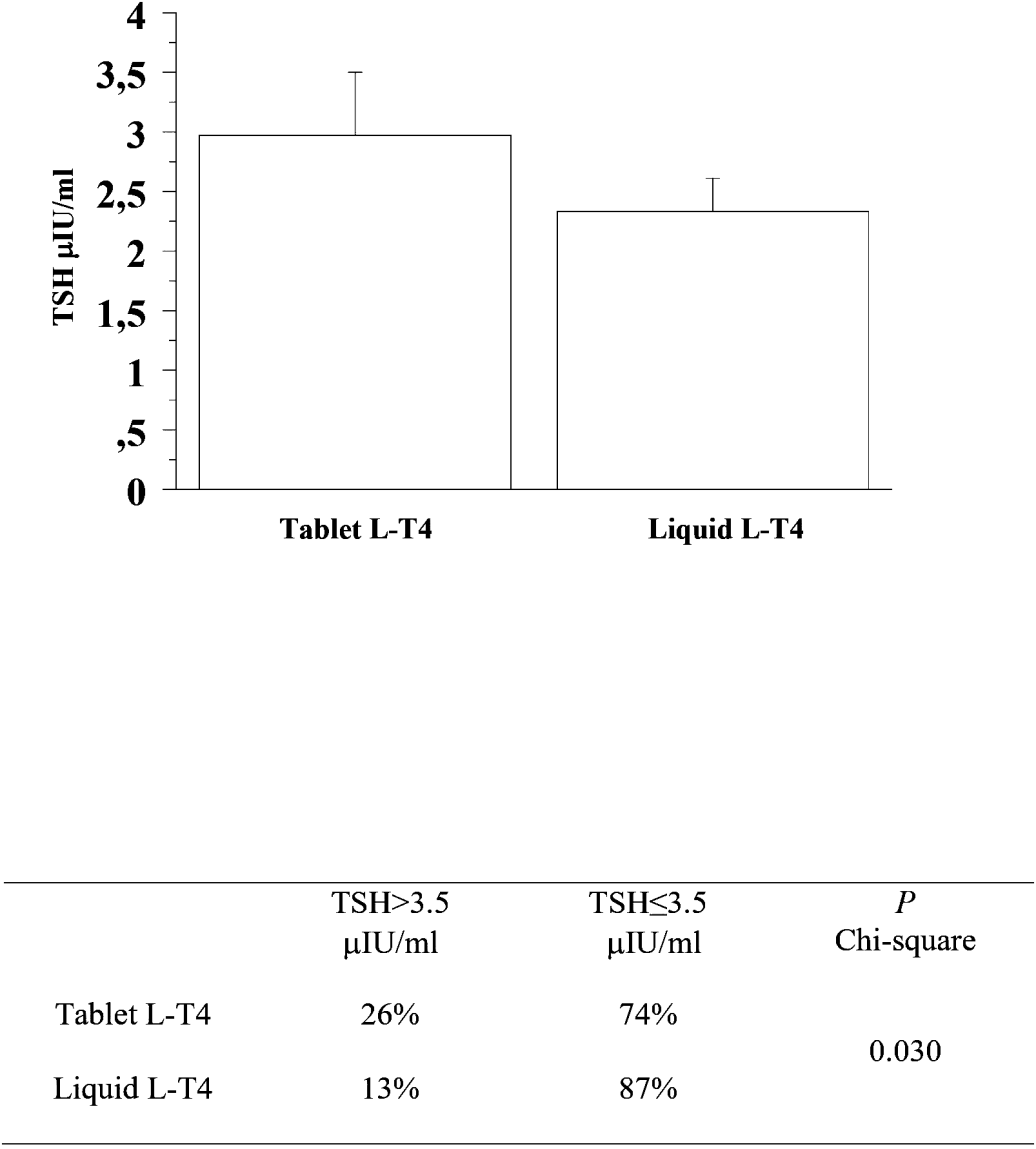
At the second re-evaluation, the prevalence of patients with a TSH higher than 3.5 μIU/mL in the T-LT4 group was 26% that was significantly higher than the prevalence in the L-LT4 group (13%). Also mean TSH level was higher, even if not significantly in the T-LT4 group with respect to L-LT4 group (ANOVA p value = 0.345).

At the second re-evaluation, the prevalence of patients with a TSH higher than 3.5 μIU/mL in the T-LT4 group was 26% that was significantly higher than the prevalence in the L-LT4 group (13%) (see **Figure 4**, **Table 2**). Also mean TSH level was higher, even if not significantly in the T-LT4 group with respect to L-LT4 group (see **Figure 4**). The prevalence of TSH levels >10 μIU/mL was 5.2% in the T-LT4 group versus 2.6% in the L-LT4 group (chi-square, p=0.37). The prevalence of patients with TSH<0.3 μIU/ml was 18.9% in the T-LT4 group against 9.1% in the L-LT4 group (chi-square, p=0.06). Mean FT4 and FT3 circulating levels were not significantly different in the two groups.

**Figure 5:**
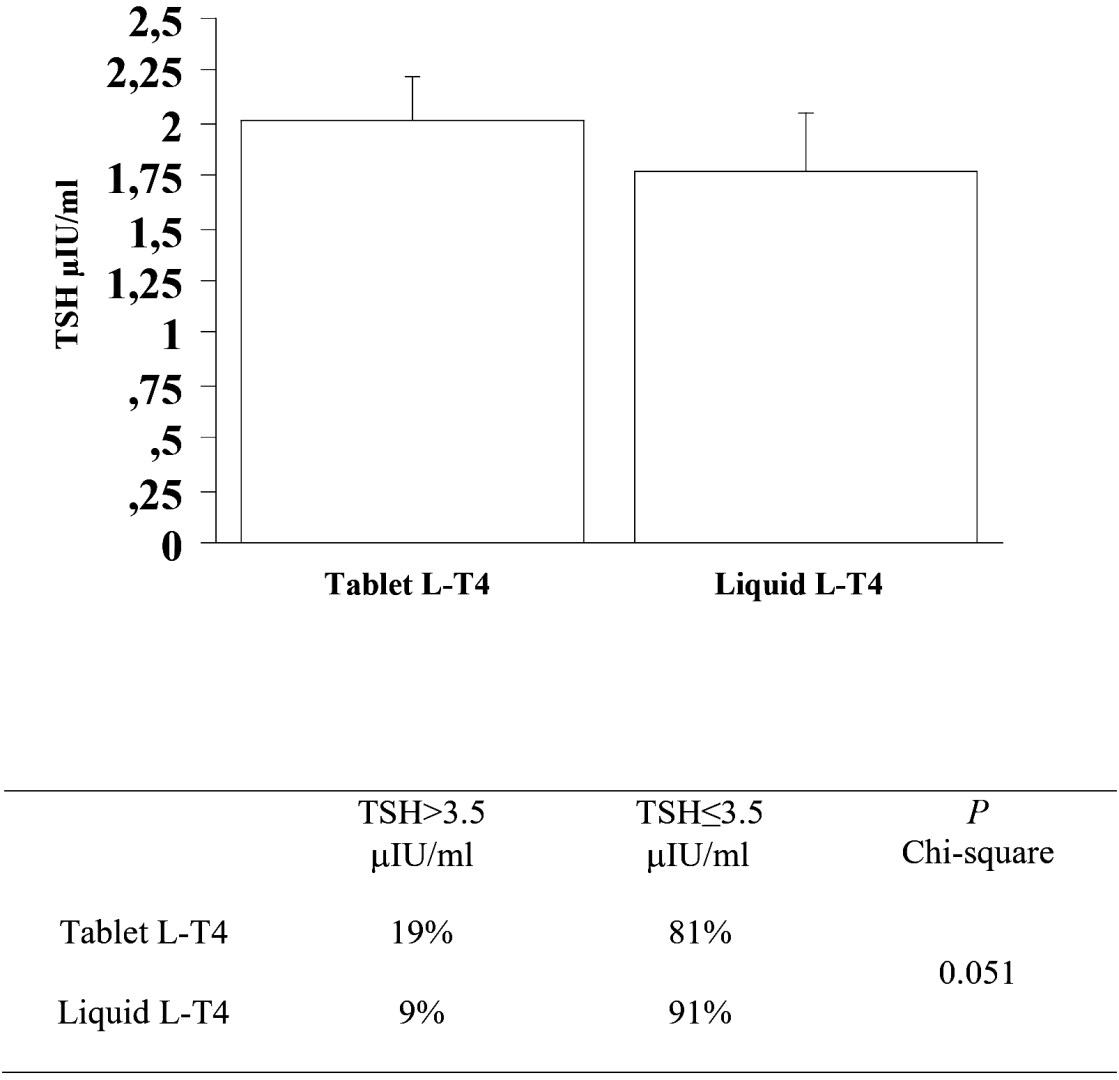
At the third re-evaluation, the prevalence of patients with a TSH higher than 3.5 μIU/mL in the T-LT4 group was 19% that was higher than the prevalence in the L-LT4 group (9%) with a statistical result near to be significant. Also mean TSH level was higher, even if not significantly in the T-LT4 group with respect to L-LT4 group (ANOVA p value = 0.491).

At the third re-evaluation, the prevalence of patients with a TSH higher than 3.5 μIU/mL in the T-LT4 group was 19% that was higher than the prevalence in the L-LT4 group (9%) with a statistical result near to be significant (see **Figure 5**, **Table 2**). Also mean TSH level was higher, even if not significantly in the T-LT4 group with respect to L-LT4 group (see **Figure 5**). The prevalence of TSH levels >10 μIU/mL was 0.98% in the T-LT4 group versus 1.44% in the L-LT4 group (chi-square, p=0.77). The prevalence of patients with TSH<0.3 μIU/ml was 16.6% in the T-LT4 group against 17.3% in the L-LT4 group (chi-square, p=0.90). Mean FT4 and FT3 circulating levels were not significantly different in the two groups.

**Figure 6:**
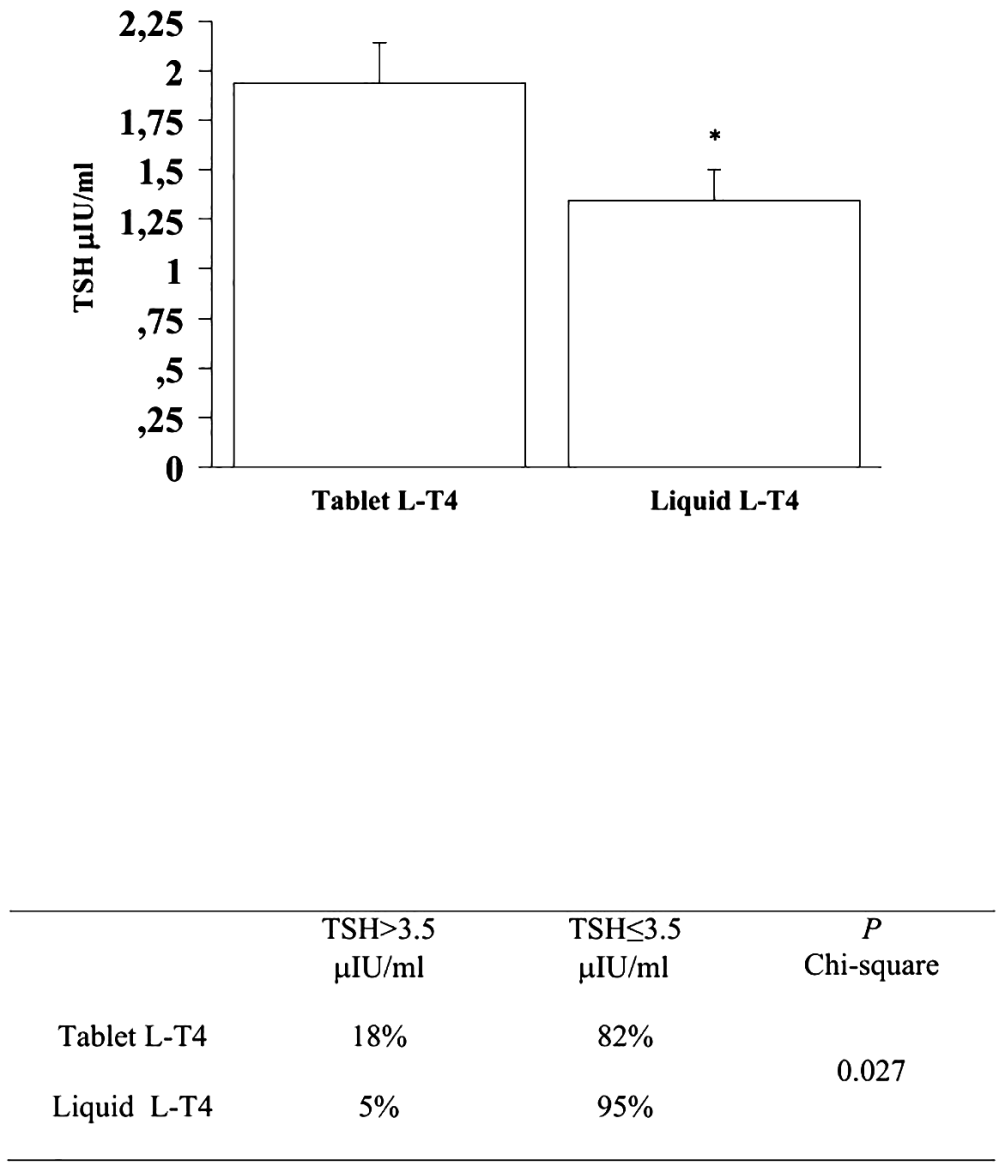
At the fourth and last re-evaluation, the prevalence of patients with a TSH higher than 3.5 μIU/mL in the T-LT4 group was 18% that was significantly higher than the prevalence in the L-LT4 group (5%). Also mean TSH level was significantly higher, in the T-LT4 group with respect to L-LT4 group (ANOVA p value = 0.034).

At the fourth and last re-evaluation, the prevalence of patients with a TSH higher than 3.5 μIU/mL in the T-LT4 group was 18% that was significantly higher than the prevalence in the L-LT4 group (5%) (see **Figure 6**). Also mean TSH level was significantly higher, in the T-LT4 group with respect to L-LT4 group (see **Figure 6**). The prevalence of TSH levels >10 μIU/mL was 2.1% in the T-LT4 group versus 0% in the L-LT4 group (chi-square, p=0.25). The prevalence of patients with TSH<0.3 μIU/ml was 15.0% in the T-LT4 group against 16.9% in the L-LT4 group (chi-square, p=0.75). Mean FT4 and FT3 circulating levels were not significantly different in the two groups.

The mean dosage increments of L-T4 to reach normal TSH values was higher even if not significantly in the tablet group (T-LT4 is 0.45 +/- 0.27 μg/kg/die, L-LT4 0.31 +/-0.22 μg/kg/die; p>0.5).

The patients during the evaluation did not present the appearance of other gastric or intestinal disorders with respect to the basal evaluation and were not treated with drugs interfering with L-T4 therapy, with exception of PPI.

## 4 Discussion

The results of the present study, that was specifically aimed to evaluate the stability of TSH and thyroid hormone levels in patients treated with different formulations of L-T4, show that at the first re-evaluation the prevalence of hypothyroidism was similar in the two groups, however, during the follow-up, at the second and fourth (and last) re-evaluations, a significantly higher prevalence of hypothyroid patients (TSH>3.5 μIU/mL) was observed in the T-LT4 group respect to the L-LT4 sample (chi-square, p=0.03 and p=0.027, respectively), with a result near to be significant at the third follow-up assessment (chi-square, p=0.051). These data suggest that the liquid L-T4 formulation therapy can result in a more stable control of TSH levels in hypothyroid patients with gastric disorders in the long-term follow-up. We have found that FT3 and FT4 circulating levels were not significantly different between the two groups during the different controls. This is absolutely in line with the results of many other studies and of our studies that evaluated the efficacy of different formulations of L-T4. In fact, TSH levels are more sensitive than thyroid hormone levels to evaluate the hormonal homeostasis in patients with hypothyroidism (13).

About 20%–50% of patients on T-LT4 do not attain a normal TSH in cross sectional studies (20) and need an adjustment of therapy (21), due to various interfering issues (22); this is in line with our results (13, 20–22).

Furthermore, the correct acidic gastric pH is mandatory for the dissolution phase of sodium L-T4 salts and their release in order to be absorbed when they reach enteric mucosa. The liquid oral solution administered in this study showed a faster absorption time in specific pharmacokinetic analysis that confirmed that it does not need the acid dissolution phase but it can directly pass through gut mucosa (area under the curve from 0 to 2 h about 50% wider; time to maximum concentration faster by a mean of 30 min), and overall better pharmacokinetics profile (14). Moreover the oral solution considered in the present study contains ethanol, an alcohol, that could further facilitate the absorption of the compound in the highly vascularized mucosa of the upper digestive tracts, such as the oral mucosa, allowing a rapid passage into the systemic circulation, although this mechanism still remains to be elucidated (23, 24). Moreover, the bioequivalence of the liquid and oral formulations according to the FDA criteria was also confirmed in a recent trial performed on a euthyroid and healthy population (25).

This advantage of the liquid formulation of not requiring the gastric dissolution phase in an acidic environment, has suggested its use in numerous subsets, in which the ingestion of the solid form may be hindered, or for anatomical reasons, as in the case of patients dysphagic, or due to the difficulty of complying with the rules that guarantee correct absorption: intake in the morning, on an empty stomach, at least half an hour before breakfast and away from foods such as coffee (26–30). With these premises, the greater compliance of the oral solution, compared to the solid form, has been studied and evaluated in several clinical settings, such as in patients undergoing thyroidectomy for both benign and malignant pathologies (31), in patients with and without different enteric malabsorption syndromes (19, 32–42) and in patients suffering from morbid obesity who have been submitted to various types of bariatric surgery (43–45).

More than 15 years ago, the first clinical evidence began to emerge of the need for the physiological gastric acid environment for the correct absorption of L-T4 tablets and therefore for achieving the desired TSH values. In fact, Centanni et al. reported that 113 patients with euthyroid multinodular goiter and suffering from chronic H. Pylori infection or chronic gastritis, required significantly higher doses of L-T4 compared to a control group of 135 patients, suffering from the same thyroid disease but not from gastric disorders, to obtain the same target TSH level (0.05 to 0.20 mU/L) (5). Based on this evidence, several years later, a study evaluated the impact of the liquid and solid solution of L-T4 in a group of hypothyroid patients, naive to hormone therapy, suffering from H.Pylori infection: TSH values were significantly lower in those treated with the liquid formulation compared to those taking the tablet, while this difference did not emerge in a second control arm of the study, which included hypothyroid patients in whom any form of gastric pathology was excluded (46). In our study, the prevalence of Helicobacter Pylori infection was the same in two groups (17% in both T-LT-4 and L-LT4 groups; p>0.05).

Furthermore, 134 of the studied patients (among a total of 204) (74 and 60 in the T-LT4 and L-LT4 groups, respectively) suffered from chronic gastritis. Autoimmune metaplastic atrophic gastritis (AMAG) is another very common chronic gastric disease and it is frequently associated with chronic autoimmune thyroiditis, also known as Hashimoto’s thyroiditis (HT), which represents the main cause of hypothyroidism in iodine-sufficient areas of the world (2). AMAG is in fact characterized by the presence of autoantibodies directed against parietal cells which, decreasing in number, lose the capacity for acid and peptic secretion, with a subsequent hypochlorhydria/achlorhydria (47). In fact, a study demonstrated that the dose of L-T4 required for the treatment of hypothyroidism associated with HT was significantly higher in patients with parietal cell antibodies, and the dose requirement was even higher in the subset of patients who, in addition to antibody positivity, showed evidence of gastric damage at gastric endoscopy (48).

Beyond their underlying gastric disorders, seventy-nine patients (66%) in the T-LT4 group and 43 (51%) patients in the L-LT4 group, were chronically treated with PPI for the whole period of evaluation. In 2014, Vita et al. demonstrated, on a small sample of 24 patients taking chronic PPIs therapy, that the transition from the tablets to liquid form, at the same daily dosage, resulted in a significant reduction in plasma TSH levels, demonstrating that the liquid solution is not affected by the average increase in gastric pH caused by the use of PPIs (11). There is also numerous evidence regarding the alteration of the absorption of L-T4 therapy in patients taking other drugs or supplements, such as calcium and iron supplements, or bile sequestrants (12, 49). The intrinsic characteristic of the oral formulation of not requiring the gastric dissolution phase can certainly facilitate the achievement of therapeutic TSH targets even in these subsets of patients. Of course, specific trials are needed to confirm these hypotheses.

To the best of our knowledge, this is the first controlled study that evaluated liquid L-T4 in patients suffering from hypothyroidism and gastric acid secretion impairment, regardless of their etiology, in comparison to a matched group of patients on L-T4 tablet. Our data show that the use of L-T4 liquid formulation resulted in a significantly higher number of treated patients with stable TSH values within the normal range during an overall period ranging from 23 to 31 months. The importance of TSH level within the normal range is essential also to improve the quality of life of these patients (28) who are often burdened with several other diseases and therapies, which could hamper a correct management of their hypothyroidism. For this purpose, it could be also noteworthy a potential reduction of blood sampling to monitor thyroid function if the chosen L-T4 formulation (i.e. liquid) can ensure a more predictable and stable effect on TSH levels.

The main limits of our study are the monocentric and retrospective nature of the performed analysis, even both groups of treated patients have been longitudinally followed. To improve the numerosity of the sample, the included patients have not been selected based on specific hypothyroidism or gastric impairment etiology. Moreover, differently from other study (46), we considered patients already on L-T4 therapy and not naïve to it. All these decisions were also driven by the aim to better represent the real-life clinical practice and the general population.

In conclusion, on the whole, our data support a better control of TSH values in hypothyroid gastropathic patients in liquid L-T4 regimen, with respect to L-T4 in tablets.

## Data availability statement

The original contributions presented in the study are included in the article/supplementary material, further inquiries can be directed to the corresponding author/s.

## Ethics statement

The studies involving humans were approved by Comitato Etico di Area Vasta Nord Ovest. The studies were conducted in accordance with the local legislation and institutional requirements. The participants provided their written informed consent to participate in this study.

## Author contributions

PF: Writing – review & editing, Writing – original draft, Formal analysis, Data curation. FR: Writing – review & editing, Writing – original draft, Formal analysis, Data curation. AP: Writing – review & editing. VM: Writing – review & editing. CB: Writing – review & editing. GE: Writing – review & editing. EBal: Writing – review & editing. EBar: Writing – review & editing. LR: Writing – review & editing. EP: Writing – review & editing. MC: Writing – review & editing. GV: Writing – review & editing. SU: Writing – review & editing. SB: Writing – review & editing. SMF: Writing – review & editing, Writing – original draft, Formal analysis, Data curation. AA: Writing – review & editing, Writing – original draft, Formal analysis, Data curation.
